# Childhood Obesity and Obstructive Sleep Apnea

**DOI:** 10.1155/2012/134202

**Published:** 2012-08-22

**Authors:** Indra Narang, Joseph L. Mathew

**Affiliations:** Division of Respiratory Medicine, The Hospital For Sick Children, 555 University Avenue, Toronto, ON, Canada M5G 1X8

## Abstract

The global epidemic of childhood and adolescent obesity and its immediate as well as long-term consequences for obese individuals and society as a whole cannot be overemphasized. Obesity in childhood and adolescence is associated with an increased risk of adult obesity and clinically significant consequences affecting the cardiovascular and metabolic systems. Importantly, obesity is additionally complicated by obstructive sleep apnea (OSA), occurring in up to 60% of obese children. OSA, which is diagnosed using the gold standard polysomnogram (PSG), is characterised by snoring, recurrent partial (hypopneas) or complete (apneas) obstruction of the upper airway. OSA is frequently associated with intermittent oxyhemoglobin desaturations, sleep disruption, and sleep fragmentation. There is emerging data that OSA is associated with cardiovascular burden including systemic hypertension, changes in ventricular structure and function, arterial stiffness, and metabolic syndromes. Thus, OSA in the context of obesity may independently or synergistically magnify the underlying cardiovascular and metabolic burden. This is of importance as early recognition and treatment of OSA in obese children are likely to result in the reduction of cardiometabolic burden in obese children. This paper summarizes the current state of understanding of obesity-related OSA. Specifically, this paper will discuss epidemiology, pathophysiology, cardiometabolic burden, and management of obese children and adolescents with OSA.

## 1. Introduction

The epidemic of pediatric obesity has caused serious concern all over the world as the prevalence has increased alarmingly over time, not only in developed countries but also in developing countries [1, 2]. Furthermore, there is increasing recognition that childhood obesity is occurring at progressively younger ages [3]. Recent publications have highlighted the challenge of defining childhood obesity in a manner that is both evidence based as well as uniformly applicable across different settings [4]. In general, a statistical definition using BMI for age is used wherein >85th percentile is defined as overweight and >95th percentile as obesity [5]. In contrast, the WHO defines obesity as BMI for age *Z*-score >3 and overweight as *Z*-score >2. A large-scale multicentric study calculated BMI in children and adolescents and extrapolated the cut-off values for adult obesity (BMI > 30) and overweight (BMI > 25), to the corresponding values in childhood and adolescence. Based on this, they were able to tabulate age- and gender-specific cut-off values for children and adolescents [6]. At the present time, waist circumference is not used routinely to define obesity in children and adolescents.

The high prevalence of obesity is believed to be a complex interplay of genetic, environmental (life-style), socioeconomic, cultural, and psychological factors which are beyond the scope of this paper. However, interestingly the pattern of in utero growth may program the pattern of subsequent body fat deposition and neuroendocrine interactions that promote eating behavior. Specifically, there is an observed increase in childhood obesity with increasing birth weight [7]. Counterintuitively, infants with low birth weight and an early adiposity rebound are also predisposed to higher rates of obesity in later childhood [8].

There are several well-documented adverse consequences of childhood obesity. Specific morbidities associated with obesity include hypertension, left ventricular abnormalities, insulin resistance, type 2 diabetes, dyslipidemia, nonalcoholic fatty liver disease, and obstructive sleep apnea [9]. Further, in obese individuals, the clustering of dyslipidemia, hypertension, and impaired glucose tolerance/insulin resistance is referred to as the Metabolic Syndrome (MetS), which further increases the risk of atherosclerotic heart disease [10]. In a followup of over 200,000 Norwegian adolescents, the relative risk of death due to ischaemic heart disease for those with a BMI > 85 percentile was 2.9 for males and 3.7 for females when compared to those with lower BMI percentiles [11]. These may underrepresent the true cardiovascular burden in adulthood given the knowledge that 75% of obese children will become obese adults [12, 13]. Additional complications of obesity include menstrual problems and polycystic ovarian disease, gallstones, orthopedic issues, and psychological stress compounding poor quality of life [14, 15] in these children.

## 2. Obstructive Sleep Apnea (OSA)

During sleep in normal individuals, there is reduction in the tone of airway musculature; however pharyngeal dilator activity keeps the airway patent. Therefore, although normal children can have occasional pauses in breathing for up to 10–15 seconds, there is no significant airflow limitation. Therefore paO_2_ may fall only by 2–4 mm Hg, and end-tidal CO_2_ may increase marginally by 3-4 mm Hg. More importantly, there is no arousal from sleep [16, 17].

Obstructive sleep apnea (OSA) is part of the spectrum of clinical conditions comprising sleep-disordered breathing (SDB). The spectrum of SDB ranges from partial to complete upper airway obstruction. In children, obstructive apnea is defined by the absence of nasal airflow despite the presence of chest wall and abdominal wall movements, for a duration of at least two breaths. In contrast, the term “obstructive hypopnea” refers to decrease in nasal airflow by 50% from the baseline accompanied by fall in oxygen saturation of 3% and/or arousal. The number of apneic and hypopneic events per hour of sleep is expressed as apnea/hypopnea index (AHI) on polysomnography [18]. In adults, AHI < 5/hour is considered normal. However, in children, AHI > 1event/hour is regarded abnormal. In general, the same criteria can be used for adolescents in the age group 12–15 years. For those beyond 18 years, it is recommended that adult criteria be used. In children, AHI is also used to categorize the severity of OSA; AHI up to 1.5 events/hr is classified as mild, 1.5–5.0 events/hr as moderate, and >5.0 events/hr as severe. This is in contrast to adults, where the corresponding values are 5–15 events/hr, 15–30 events/hr, and >30 events/hr.

OSA is characterised by snoring, recurrent partial (hypopneas), or complete (apneas) obstruction of the upper airway. OSA is associated with intermittent oxyhemoglobin desaturation, sleep disruption, and fragmentation [19]. About 3–12% of the “healthy” pediatric population has habitual snoring, whereas only 1–3% has OSA [20, 21]. However it should be noted that snoring is not synonymous with OSA. Habitual snorers typically do not have obstructive apnea, hypopnea, respiratory effort-related arousals, or abnormal gas exchange. This is because neuromuscular compensation in these children prevents significant airway obstruction.

In OSA, the episodes of airway obstruction can be related to increased airway collapsibility on account of mechanical and neuronal factors. The most common mechanical factor in children is hypertrophy of adenoids and/or tonsils narrowing the airway lumen [22]. Approximately 2% of otherwise healthy children have large tonsils and adenoids that mechanically obstruct airways [20, 23]. However, OSA is a balance of mechanical obstruction and decreased activity of pharyngeal dilator muscle activity. During sleep, children with OSA have reduced airway muscle tone which critically narrows and obstructs the airway, resulting in upper airway obstruction. The consequent hypoxemia results in arousal with restoration of airway tone and relief of the obstruction. The frequency of episodic apnea determines the diagnosis and severity of OSA.

## 3. Diagnosis of OSA

OSA can be suspected by the presence of both nocturnal as well as day-time symptoms. The most common nighttime symptoms are snoring during sleep; sometimes parents are able to describe characteristic episodic pauses in breathing despite movement of the chest or abdomen. Other descriptions include gasping, restlessness during sleep, nighttime sweating, sleeping in unusual positions, parasomnias (sleep terrors, sleep walking), and secondary nocturnal enuresis.

 The daytime symptoms are based on functional consequences of disturbed sleep and/or hypoxemia/hypercarbia. These include early morning headache and sometimes nausea or vomiting, excessive daytime sleepiness, and fatigue. Recent reports have also highlighted neurocognitive consequences of OSA including decreased concentration, diminished memory, difficulty in making decisions, learning difficulties, and also behavioural manifestations such as hyperactivity mimicking ADHD, unusual aggressiveness, and even social withdrawal.

Children with OSA are often mouth breathers and sometimes have hyponasal speech. Children with severe OSA can also have growth stunting. Clinical examination usually reveals a crowded oropharynx, enlarged tonsils, and reduced peritonsillar space. Sometimes, a large tongue may also contribute to airway obstruction. Endoscopic examination identifies hypertrophied adenoids.

Currently, polysomnography (PSG) or a sleep study is the gold standard for a specific diagnosis of OSA [24]. Given the complexity of this investigation in terms of skill, resources, and time, some investigators have tried to use alternate approaches such as clinical questionnaires, objective and/or subjective measures of daytime sleepiness, overnight oximetry, audio recording, video recording, and nap polysomnography (during the daytime). While some of these methods can identify children with OSA, they have poor negative predictive value [25].

## 4. Obesity and Obstructive Sleep Apnea

There is now ample data confirming that OSA associated with obesity is highly prevalent in children and adolescents. The association between obesity and OSA emerges from two sets of observations; the first is the observed high prevalence of OSA among obese children and adolescents, and the second is the higher proportion of children with OSA who are obese. Thus it appears that both conditions can coexist and yet potentiate the adverse impact of each. It is believed that the prevalence of OSA among obese children and adolescents can be as high as 60% [26]. In one study of obese children undergoing polysomnography, it was observed that 46% had OSA [27]. Likewise, in another study OSA was observed in 59% of obese children undergoing evaluation for symptoms of sleep-disordered breathing [28]. Yet another study reported that 55% children scheduled for bariatric surgery for morbid obesity had OSA [29]. In fact, in many children and adolescents with OSA, the severity of OSA parallels the severity of obesity [30]. A recent population-based study involving 400 children between 2 and 8 years of age found that obesity was the most significant risk factor for OSA with an odds ratio of 4.69 (95% CI 1.58–13.33). Further analysis suggested that for each unit increase in BMI, there was a 12% higher risk of OSA [23].

## 5. Mechanisms for Increased Risk of OSA in Obese Children and Adolescents

There are multiple factors that interact to significantly increase the risk of OSA among obese children and adolescents.

Similar to nonobese children, airway obstruction by adenotonsillar hypertrophy is a fairly common cause of OSA among obese children [31–33] affecting approximately 45% of all obese children with OSA [34]. However, alarmingly, following adenotonsillectomy, OSA persists in about 50% of obese children [35] which is significantly higher than the observed persistence rate of 10–20% amongst nonobese children [36, 37]. Another additional interesting observation is that the prevalence of adenotonsillar hypertrophy among obese children is higher than among nonobese children, which indirectly suggests that adenotonsillar hypertrophy in obese children could be a consequence of another distinct mechanism. Possible explanations include endocrine mediated somatic growth that results in larger and/or heavier fat pads, soft palate, and tongue among adults with obesity [38]. It is possible that similar mechanisms operate in children and adolescents as well.

Functional factors that operate to promote upper airway obstruction OSA in obese individuals during sleep include altered neuromuscular tone resulting in greater upper airway collapsibility during sleep. Indeed, measurements of airway flow and mechanics have shown that in obese children, there is a positive critical closing pressure of the pharynx causing the airway to collapse during sleep with even mild negative inspiratory pressure [39].

Additional mechanical factors that predispose to functional abnormalities include central adiposity and an excess mechanical load on the chest wall. These factors interact and result in decreased chest wall excursion and decreased diaphragmatic excursion causing a reduction in, chest wall compliance with reduced functional residual capacity and tidal volumes. As a result, hypoventilation, atelectasis, and ventilation/perfusion mismatch may ensue resulting in increased work of breathing resulting in fatigue, all of which may be exacerbated during sleep and could further predispose to sleep-disordered breathing. Although these interacting physiologies are not well understood, they could in part explain why adenotonsillectomy is not curative in all obese children with hypertrophied adenoids and tonsils.

## 6. Obesity, OSA, and Associated Comorbidities in Obese Children and Adolescents

### 6.1. Excessive Daytime Sleepiness (EDS)

 EDS is prevalent among obese children with and without OSA [40]; specifically EDS increased progressively and significantly with increasing BMI. Prepubertal obese subjects with OSA have more EDS than non-obese subjects with OSA of similar severity [41].

### 6.2. Quality of Life (QOL)

Multiple published studies demonstrate reported poor QOL among overweight and obese children and adolescents [42] and those with OSA [43]. In one study with 151 children, with a mean age of 12 years, the presence of OSA was a predictor of poor QOL in overweight children [44].

### 6.3. Neurocognitive Function

 OSA is associated with cognitive, behavioral, and functional deficits in young children [45]. Although total sleep duration may modulate behavioral function, it is believed that sleep fragmentation associated with OSA is a key determinant of behavioral alterations in pediatric OSA subjects. A recent study with 52 children reported improvement in both neurobehavioral function and daytime sleepiness in children who used an average of 3-hour positive airway pressure (PAP) at night [46]. In another small study with 6 obese adolescents, even modest level of PAP adherence displayed improved attention and school performance whereas a similar group of 7 nonadherent adolescents showed academic decline [47]. Resolution of OSA is associated with improvement in neurocognitive status.

### 6.4. Physical Activity

Physical activity levels are reduced both in obese children and those with OSA [48]. Increased physical activity may not only promote weight loss but also, secondary to weight loss, may improve the severity of OSA [49].

### 6.5. Cardiovascular Burden

Multiple adult studies indicate that OSA contributes to or exacerbates cardiovascular disease in the context of obesity [50]. A similar evaluation of childhood obesity-related OSA on cardiovascular structure and function is currently not available. However, indirect measurements that reflect blood pressure regulation, cardiac function, autonomic dysfunction, and endothelial properties suggest a similar pattern in obese children and adolescents [48, 51, 52]. The precise mechanisms linking cardiovascular disease both to OSA and obesity are not completely understood. However, a common mechanism is activation of the sympathetic nervous system. Specifically, repetitive arousals, episodic hypoxaemia, hypercapnia, and changes in intrathoracic pressures lead to sympathetic activation via chemoreceptor activation, impaired baroreflex sensitivity, and increased activity of the renin-angiotensin system. In obesity, increased adiposity elevates levels of free fatty acid (FFA) which with increased levels of leptin promote sympathetic activation. Chronic sympathoactivation instigates dyslipidaemia, left ventricular modelling, endothelial dysfunction and arterial stiffness, inflammation with high levels of hs-CRP, and insulin resistance with resultant glucose intolerance. All of these factors are inextricably linked and together induce significant cardiovascular morbidity. In addition, episodic hypoxemia in children with OSA causes pulmonary vasoconstriction and ultimately pulmonary artery hypertension [49, 53].

**Hypertension**

In children, obesity is a risk factor for high BP, and OSA is independently associated with increased BP [51, 54]. In a recent study, among prepubertal, non-obese children, the presence of OSA was associated with an elevation in BP by 10–15 mmHg independently of BMI during both wakefulness and sleep when compared to nonsnoring controls (ZA). In a separate study of 140 children, children with severe OSA when compared with controls with no OSA showed significantly increased mean arterial BP during awakefulness and sleep, increased diastolic BP during wakefulness and sleep, and increased systolic BP during sleep. Almost one-third of the patients with severe OSA showed a mean 24-hour systolic BP > 95th percentile. Similarly, obese children with moderate-to-severe OSA had a significantly increased risk for hypertension than obese children with mild OSA, suggesting that OSA may be a trigger for hypertension in obese children [55]. These findings are of significance as a recent longitudinal study has shown that elevated BP in childhood tracks into adult life and is associated with an increased risk of hypertension and metabolic syndrome later in life [56].

**Changes in Ventricular Structure and Function**

In adulthood, there is a significant association between left ventricular mass and cardiovascular mortality. Pilot data shows a significantly higher left ventricular mass index (LVMI) with reduced diastolic and systolic function among obese children without documented OSA compared with lean controls [57]. In non-obese children with OSA, abnormalities in LVMI correlate with both the presence and severity of OSA [58]. One study reported that subjects with severe OSA had an odds ratio of 11.2 for LVMI > 95th percentile [59], while another showed that relief of OSA following an adeno-tonsillectomy resulted in measured cardiac variables in the same range as controls [58]. Furthermore, in non-obese children with OSA, improvements in LV diastolic function [59] and the right ventricular myocardial performance index have been observed after resolution of OSA [60]. Thus OSA in the context of obesity is likely to exacerbate abnormalities of LV structure and function.

**Endothelial Function**

OSA is also involved in causing endothelial dysfunction, mediated by reduced levels of nitric oxide and increased levels of mediators like endothelin-1 and plasma aldosterone.

**Cardiac Autonomic Activity**

Cardiac autonomic activity is usually measured using indices of heart rate variability (HRV). Low HRV signifies sympathetic overdrive and has been consistently associated with the risk of incident cardiovascular disease in adults. In obese children, HRV was lower than non-obese children with body weight as the strongest predictor for lower HRV [61]. However, in non-obese children with moderate-to-severe OSA, HRV variability was lower compared to those without OSA [62].

### 6.6. OSA and the Metabolic Syndrome

There is emerging data that OSA itself mediates insulin resistance, dyslipidemia, hypertension, and inflammation. These occur through sympathetic hyperactivity, intermittent hypoxemia, and sleep fragmentation or insufficient sleep. In other words, OSA may be a cause of obesity and not a consequence alone. This conclusion stems from independent pieces of evidence that point towards the contribution of OSA to various components of the metabolic syndrome and perhaps, more importantly, the reversibility with treatment of OSA.

Recent data showed increased levels of insulin (indicating insulin resistance) in adolescents with OSA [63]. In younger children, adeno-tonsillectomy is associated with improvement in lipid profile, insulin sensitivity, and inflammatory markers in some studies [64, 65].

### 6.7. Contribution of OSA to Obesity

OSA is associated with inadequate sleep quantity and quality in children as well as adults. A recent systematic review [66] examining the relationship between sleep duration and the development of obesity reported that in children and adults, shorter duration of sleep was associated with increased risk of obesity (odds ratio 1.89, 95% CI 1.46–2.43 in children). Thus OSA can have a direct impact by worsening obesity.

## 7. Management

It is clear that childhood obesity and OSA can present to a wide range of professional disciplines on account of the multisystem manifestations. Therefore successful management depends on concerted effort by a multidisciplinary team of professionals including sleep physician, ENT surgeon, respirologist, child nutritionist, child psychologist, cardiologist, and social worker, working together with the obese child and his/her family. The goals of management are enhanced quality of life and prevention of short- and long-term complications.

## 8. Management of Obesity

A detailed discussion on the various modalities for weight reduction and management of obesity is outside the scope of this paper; however a brief review of the current recommendations is presented. Although the body of evidence in children and adolescents is still being generated, it is generally recommended they should perform at least 60 minutes of moderately intense physical activity daily, in order to prevent obesity or maintain weight. This should be encouraged even if it does not result in weight loss, on account of the general health benefits of exercise. They should also be advised to eat meals at regular times and preferably free from distractions.

Pharmacological interventions are generally not recommended for children below 12 years, barring exceptional circumstances such as severe OSA or raised intracranial tension. In addition, the rare decision to use pharmacotherapy does not exclude the need for physical activity and dietary control. Some experts maintain that medication is better utilized to maintain weight loss, rather than induce it. Currently, Orlistat is the only pharmacological agent that can be considered, since Sibutramine has been withdrawn.

Bariatric surgery is also rarely recommended in children, unless they are morbidly obese (BMI ≥ 40 kg/m^2^) or 35–40 kg/m^2^ with coexisting diseases that could be improved by loss of weight. Even then, surgery is considered only after nonsurgical measures have been tried without success.

## 9. Management of OSA

Based on the observation that almost half of all obese children with OSA have adeno-tonsillar hypertrophy, the American Board of Pediatrics [24] recommends adeno-tonsillectomy as the first step in management. Although adeno-tonsillectomy results in improvement in 80% cases and improves obstructive symptoms in 80% of cases of otherwise normal children with OSA, children with morbid obesity are more likely to fail treatment than normal children. In some series, almost 50% continue to have OSA [55].

Therefore Positive Airway Pressure (PAP) therapy has become the standard of care, usually in conjunction with weight loss strategies. In adult patients, PAP therapy results in dramatic improvement in OSA symptoms. In addition, there are encouraging reports of improvement in cardiovascular status including reduction in systolic and BP, LV function [67], markers of endothelial function [68], and cardiac autonomic activity [69]. In addition, withdrawal of PAP for two weeks was associated with systolic and diastolic BP increase of 4–6 mmHg [70]. PAP therapy can be administered either as continuous PAP (CPAP) or the more physiological bilevel PAP (BiPAP). In children also, the symptoms of OSA improve with PAP therapy; however there is limited data evaluating its efficacy in improving clinical outcomes and QOL.

In one study, non-obese children who had resolution of OSA, 6 months following adeno-tonsillectomy, had a reduction in diastolic BP of the order of 5 mmHg [71]. The importance of this study cannot be overemphasised in the context of recent findings of a large meta-analysis that lowering systolic SBP by 10 mmHg or diastolic BP by 5 mmHg in adults (regardless of the baseline BP) reduced fatal and nonfatal cardiac events by approximately 25% [72].

Other treatment options that are sometimes useful in adult patients with OSA include oral appliances and devices that expand the upper airway space. However these require skilled construction and are generally efficacious in mild OSA only. However it is a viable option for those who cannot or will not use CPAP. These appliances have limited value in children on account of less developed dentition [73]. Some adults use simple devices to prevent sleeping in the supine position. These devices work by promoting sleep in the lateral or prone position.

Surgical management options include uvulo-palatopharngoplasty wherein bulky soft tissues that obstruct the airway can be trimmed or excised to create a larger airway space. It has also been used to strengthen and support hypotonic pharyngeal muscles in those children where reduced neuromuscular tone is responsible for airway floppiness and obstruction. Some centres use the procedure for obese children with severe OSA, to reduce redundant oropharyngeal tissue bulk.

 Presently, there is no randomized trial comparing the various modalities in children adolescents, to estimate the superiority of one over the other.

## 10. Conclusion

 Childhood and adolescent obesity have reached epidemic proportions worldwide.

OSA significantly complicates obesity and is an independent risk factor for cardiovascular, metabolic, neuro-cognitive burden as well as negative impact on the quality of life in obese children.

All disciplines involved in the well-being of obese children must be involved in sleep surveillance strategies to highlight obese children at an increased risk for OSA. Early recognition and treatment of OSA, in addition to weight loss strategies, could provide an opportunity for cardiovascular and metabolic risk reduction in childhood which would positively impact the health of these children not only in childhood but also in adulthood.
